# Hydrotalcite-Modified Clinoptilolite as the Catalyst for Selective Catalytic Reduction of NO with Ammonia (NH_3_-SCR)

**DOI:** 10.3390/ma15227884

**Published:** 2022-11-08

**Authors:** Agnieszka Szymaszek-Wawryca, Paulina Summa, Dorota Duraczyńska, Urbano Díaz, Monika Motak

**Affiliations:** 1 Faculty of Energy and Fuels, AGH University of Science and Technology, Al. Adama Mickiewicza 30, 30-059 Krakow, Poland; 2 Jerzy Haber Institute of Catalysis and Surface Chemistry, Polish Academy of Sciences, Ul. Niezapominajek 8, 30-239 Krakow, Poland; 3 Instituto de Tecnología Química, UPV-CSIC, Universitat Politècnica de València-Consejo Superior de Investigaciones Científicas, Avenida de los Naranjos, s/n, 46022 Valencia, Spain

**Keywords:** hydrotalcite, clinoptilolite, co-precipitation, iron, copper, mixed oxides, DeNO_x_

## Abstract

A series of clinoptilolite-supported catalysts, modified with hydrotalcite-like phase (HT) by co-precipitation, were prepared and tested in NH_3_-SCR reactions. It was found that deposition of HT on clinoptilolite increased conversion of NO within 250–450 °C, and that the positive impact on the catalytic activity was independent of HT loading. The promoting effect of clinoptilolite was attributed to Brönsted acid sites present in the zeolite, which facilitated adsorption and accumulation of ammonia during the catalytic process. Concentration of N_2_O in the post-reaction gas mixture reached its maximum at 300 °C and the by-product was most likely formed as a consequence of NH_4_NO_3_ decomposition or side reaction of NH_3_ oxidation in the high-temperature region. The gradual elimination of nitrous oxide, noticed as the material with the highest concentration of hydrotalcite phase, was attributed to the abundance of oligomeric iron species and the superior textural parameters of the material. UV-Vis experiments performed on the calcined samples indicated that Fe sites of higher nuclearity were generated by thermal decomposition of the hydrotalcite phase during the catalytic reaction. Therefore, calcination of the materials prior to the catalytic tests was not required to obtain satisfactory overall catalytic performance in NO reductions.

## 1. Introduction

Over the last decades, layered double hydroxides (LDHs), also called as “hydrotalcite-like anionic clays”, have attracted considerable interest from the scientific community. The increasing attention directed at the application of LDHs in many fields results from the unique structural features and chemical composition of layered doubled hydroxides, which can be easily adjusted depending on the intended use. The general formula of layered double hydroxides is [*M*_1−*x*_^II^ *M*_x_^III^ (OH)_2_] [A*_x/n_^n−^*]·*m*H_2_O, where *M*^II^ and *M*^III^ correspond to divalent and trivalent metal ions, respectively, while *A^n−^* represents non-organic or organic interlayer anions [1]. The group of LDHs can be divided into natural and synthetic anionic clays. The natural hydrotalcite reflects a brucite-like structure, in which Mg^2+^ cations are partially replaced with Al^3+^ cations. The positive charge generated by this substitution is typically balanced by carbonate anions (CO_3_^2−^), which co-exist in the interlayer space with water molecules [2]. Due to the fact that Mg^2+^ and Al^3+^ cations can be substituted by other chemical elements of similar atomic radius, a great number of LDHs with a wide range of Me^2+^/Me^3+^ combinations can be prepared [3]. Furthermore, synthetic layered double hydroxides can be obtained by various procedures, e.g., sol-gel [4], rehydration [5], induced hydrolysis [6], or co-precipitation, which is the most widespread method [1]. Despite the fact that LDHs have many applications in catalytic processes, in most cases they are precursors for synthesis of mixed oxides, rather than catalysts themselves. Thermal decomposition of LDHs in conditions above 400 °C leads to the formation of oxide products with relatively high specific surface area and uniformly dispersed metal species within the oxide matrix [7,8].

One of the possible applications of LDHs-derived materials is the selective catalytic reduction of nitrogen oxides with ammonia (NH_3_-SCR). The process aims to eliminate NO_x_ produced by stationary and mobile sources of emissions to produce the mixture of N_2_ and H_2_O [9]. In fact, the commercial catalysts of NH_3_-SCR, V_2_O_5_-MoO_3_(or WO_3_)-TiO_2_, exhibit relatively good catalytic activity; however, their performance is likewise limited, mainly due to a narrow temperature window, sensitivity to SO_2_ and alkali metals, or the emission of cancerogenic vanadium [10]. Therefore, finding a substitutive catalyst of NH_3_-SCR has become a central issue, and mixed metal oxides are among the most commonly investigated potential substitutes. The advantageous catalytic features of mixed metal oxides result mainly from their high chemical versatility, satisfactorily low-temperature activity, excellent redox properties, and synergistic interactions between the active components during the catalytic reaction [11,12,13]. Most of the research on calcined LDHs has focused on the systems containing copper, cobalt, manganese, and aluminum oxides. The early findings published by Chmielarz et al. [14] indicated that catalytic activity of Co-Cu-Mg-Al mixed oxides in NO elimination with ammonia strongly depends on the content and type of transition metal. The authors declared that the catalyst with the highest content of Cu exhibited almost 95% of NO conversion at 250 °C, thus, were very active in low-temperature NH_3_-SCR. Nevertheless, in high-temperature regions, selectivity to N_2_ decreased, mainly due to a high yield of N_2_O. Wierzbicki et al. [15] suggested that the problem of low selectivity of Cu-Co mixed oxides derived from LHDs can be solved by doping with manganese. The authors introduced a precursor of Mn during the co-precipitation of hydrotalcite or by the adsorption from a solution of Mn–EDTA complex on the prepared LDH. A positive influence on NO conversion was observed only in the case of the directly synthesized sample. However, the concentration of N_2_O emitted during the reaction over the most active catalyst was still relatively high. Considering the highly oxidizing character of Cu and Mn, the studies on the composition of LDHs for SCR were shifted into the synthesis of brucite-like structures containing other transition metals. Among them, iron has been proposed as the most appropriate, due to its excellent redox features, superior resistance against SO_2_ poisoning, non-toxicity, and low price [16]. In their 2018 study, Wang et al. [17] prepared NiFe mixed oxides via the calcination of hydrotalcite-like precursors and tested the materials in NH_3_-SCR. The authors found that calcination of the precursors in moderate conditions enhanced redox properties. Nonetheless, despite good stability and high selectivity to N_2_, satisfactory conversion of NO was obtained only within 210–330 °C. The insight into the elimination of NO_x_ using hydrotalcite-derived mixed metal oxides has been presented comprehensively in the minireview of Jabłońska and Palkovits [18]. Additionally, apart from in cases of application in NH_3_-SCR, LDHs-derived mixed metal oxides have been widely investigated as the catalysts of selective catalytic oxidation of ammonia (NH_3_-SCO) by the group of Chmielarz and co-workers [19,20,21]. 

According to studies of the literature, the major challenge in the application of LDHs in NH_3_-SCR is adjustment of the preferable composition, which would decrease the generation of by-products and improve catalytic activity in the entire temperature range of the process. Both the activity and selectivity of transition metals are strongly correlated with their speciation in the catalysts. Beneficial distribution of the active phase can be provided by the support, which can also influence the entire chemical character of the catalytic system. Thereby, deposition of hydrotalcite-like phase on the surface of an appropriate support seems to be a good solution for the problem of narrow temperature window and low selectivity. 

The potential candidate for the support of NH_3_-SCR catalyst is one of the most common natural zeolites, clinoptilolite, belonging to the heulandite (HEU) family. The general chemical formula of the material is (Na,K)_6_Al_6_Si_30_O_72_·20 H_2_O, and its deposits can be found almost worldwide [22,23]. The increasing interest in the application of clinoptilolite in industrial processes results from its noteworthy physicochemical features, such as high ion-exchange capacity and mechanical crush strength (even up to 7 MPa), combined with its low price. Therefore, clinoptilolite has been applied for many environmental processes, i.e., as an adsorbent for the purification of water [24,25,26]. It was also tested in the studies of Saramok, Szymaszek-Wawryca, and co-workers [27,28] as a potential support of the catalyst used in NH_3_-SCR unit in a nitric acid plant. Moreover, due to the natural presence of catalytically active iron in its structure, non-modified clinoptilolite was investigated by Gelves et al. [23] as a catalyst itself. However, to our knowledge, no one so far has studied NH_3_-SCR catalytic performance of LDHs precipitated on zeolites. Most research on zeolite-supported catalysts involved standard modification procedures, such as solid-state ion exchange [29], incipient wetness impregnation [30], mechanical mixing with metal oxides [31], and many others. Alternatively, the active phase can be introduced directly on the synthesis level, as described in the previous study of Szymaszek-Wawryca et al. [32,33], which concerned layered zeolites of the MWW family. However, so-called one-pot synthesis is possible only in the case of synthetic zeolites, which are generally more expensive compared to their natural counterparts. Despite the great quantity of research on the SCR catalytic performance of metal-modified zeolites, it has not been established yet if the active phase can be co-precipitated in a form of LDHs. 

Considering the above-mentioned, this study explores for the first time zeolite-supported catalysts of NH_3_-SCR, prepared by co-precipitation of LDHs containing Cu and Fe. To date, research on the application of hydrotalcite-like materials in NH_3_-SCR has been mostly focused on bimetallic LDHs containing nickel and titanium [17,34,35]. Interestingly, despite the excellent synergy between iron and copper in NO reduction by ammonia [36,37], the influence of the co-existence of Cu and Fe in the hydrotalcite-like structure on the reaction rate of NH_3_-SCR has not been established yet. Hence, in the presented work, we aimed to examine the catalytic performance of Fe-Cu LDHs, in which the approximate mass ratio of Fe:Cu was 2:1. Besides, to our knowledge, no one so far has analyzed the physicochemical and catalytic features of LDHs deposited on a zeolitic support. Thus, in order to develop ecologically friendly and cost-effective material, we used clinoptilolite as a potential carrier of the catalyst. Despite its wide availability, superior catalytic features, and relatively low cost, only several studies have explored clinoptilolite as a support of NH_3_-SCR catalysts [23,27,28]. As a consequence, the presented work provides an innovative approach for the synthesis of novel NH_3_-SCR catalysts, which exhibit physicochemical properties of layered double hydroxides and zeolites. Moreover, in contrast to the LDHs catalysts described in the literature [15,17,38], the materials obtained after co-precipitation of hydrotalcite have not been calcined prior to catalytic tests. According to the study conducted by Wu et al. [34], NO reduction over hydrotalcite-like materials strongly depends on the calcination temperature. Thereby, finding the most appropriate conditions for the thermal pretreatment of LDHs requires additional and time-consuming experiments. In order to avoid that, in contrast to the great majority of the published works, we investigated whether thermal treatment of LDHs deposited on the zeolite can be performed during the catalytic reaction. Additionally, we intended to establish the most beneficial coverage of the chosen zeolite with the hydrotalcite-like phase. All in all, the presented works provide the foundation for a new way to prepare novel NH_3_-SCR catalysts which bind the features of various materials.

## 2. Materials and Methods

### 2.1. Preparation of the Catalysts

The single-phase hydrotalcite-like material containing Al^3+^ and Fe^3+^ (as the trivalent metals, Me^3+^) and Mg^2+^ and Cu^2+^ (as the divalent metals, Me^2+^) with an Me^2+^/Me^3+^ molar ratio of 3:1 were prepared via a co-precipitation method at constant pH proposed by Cavani and co-workers [1]. Catalysts were designed to contain 5 wt% of iron and 5 wt% of Cu in the oxide form. Typically, the aqueous solution containing the mixture of metal nitrates was added dropwise into the aqueous solution of sodium carbonate at 65 °C under stirring conditions. Constant pH of 9.5–10 was adjusted by the introduction of 1 M solution of NaOH, which was added simultaneously with the mixture of metal precursors. Subsequently, the obtained precipitate was aged for 1 h at 65 °C in the mother solution. Afterwards, the formed solid was filtered, washed with the distilled water to remove the residual nitrates, and dried overnight at 80 °C. The prepared material was labeled as HT.

Preparation of clinoptilolite-supported catalysts with co-precipitated hydrotalcite phase started from the transformation of natural zeolite into the hydrogen form. The raw clinoptilolite (labeled as Clin) was protonated using 5 wt% of HCl solution. Typically, the material was introduced into the adequate volume of acid to obtain zeolite/HCl ratio of 1/3. The suspension was shaken for 2 h, filtered, and washed with distilled water. The shaking procedure was repeated three times, and afterwards the sample was dried at 100 °C in air. The obtained zeolitic support was labeled as H-Clin.

The catalysts investigated in this work were prepared by co-precipitation of hydrotalcite-like phase at constant pH directly on the surface of the protonated clinoptilolite. Firstly, 5 g of H-Clin were introduced into the aqueous solution of Na_2_CO_3_ and the suspension was kept under vigorous stirring at 65 °C. In order to enhance the adhesion of the zeolitic surface, 2 cm^3^ of propylene glycol were added dropwise into the solution. Co-precipitation of hydrotalcite was performed according to the same procedure as described for the HT sample. Due to high acidity of H-Clin, a higher amount of NaOH solution was required to maintain the desired basic pH of the reacting mixture. The amounts of metal nitrates were adjusted adequately to obtain 20, 25, and 30 wt% coverage of H-Clin with the hydrotalcite phase.

### 2.2. Characterization of the Catalysts

The chemical composition of the catalysts in relation to the content of Si, Al, Mg, Cu, and Fe was determined by inductively coupled plasma optical emission spectroscopy (ICP-OES, QTEGRA). Crystallinity and phase purity were analyzed by X-ray powder diffraction (XRD). The XRD patterns were obtained using a Panalytical Empyrean diffractometer. The diffractograms were collected using Cu Kα radiation source (λ = 1.54184 Å) at a tube current of 40 mA and a voltage of 40 kV in the scanning range of *2θ* of 3–90 °. The recorded XRD data were analyzed using X’pert HighScore software plus (with database). The size of crystals of single-phase hydrotalcite was calculated according to the Debye–Scherrer equation, including a correction related to the instrument broadening. Besides this, the unit cell parameters of the material were determined by the method proposed by Rives [39]. Textural properties and specific surface area of the materials were analyzed by low-temperature N_2_ sorption using a Micromeritics 3Flex Surface Characterization gas adsorption analyzer. Prior to the experiment, the samples were degassed under vacuum conditions at 90 °C for 1 h and afterwards, at 350 °C for 5 h. The specific surface area (*S_BET_*) of the materials was calculated using the BET (Brunauer–Emmet–Teller) model, from the adsorption branch, according to the recommendations of Rouquerol [40]. Scanning electron microscopy (SEM) studies were carried out using Field Emission Scanning Electron Microscope JEOL JSM-7500 F equipped with an X-ray energy dispersive (EDS) system. SEI images were provided by the secondary electron detector. Thermogravimetric studies (TG) were performed to determine changes in the weight of the samples within 30–800 °C, with the temperature ramp of 10 °C·min^−1^. Typically, the sample was placed on a platinum holder in the mixture of air and helium. Thermogravimetric and differential thermal analyses (TGA-DTA) consisted of progressively increasing the temperature to quantify the weight losses, which solid samples suffer. The procedure consisted of heating the sample to 800 °C with a temperature ramp of 10 °C/min using an air stream of 20 mL/min. Mettler Toledo TGA/SDTA 851E equipment was used for the analyses carried out. The characteristic chemical groups present in the materials were studied by FT-IR spectroscopy. The spectra were collected using a Perkin Elmer Frontier spectrometer in the wavelength region of 4000–400 cm^−1^, with a resolution of 4 cm^−1^. The coordination and aggregation of metal species introduced into the zeolites were investigated by ultraviolet diffuse reflectance spectra (UV-vis-DR). The analysis was carried out using a Cary 5 spectrophotometer, equipped with a diffuse reflectance accessory. The spectra were taken in the range of 200–800 nm, with a resolution of 2 nm.

### 2.3. Catalytic Tests

The catalytic performance of the prepared materials in NH_3_-SCR reaction was analyzed in a fixed-bed flow microreactor with a quartz tube under atmospheric pressure. Typically, 200 mg of the catalyst with 20–35 mesh was outgassed in a flow of nitrogen at 400 °C for 30 min. The material was cooled down to 100 °C and exposed to the gas mixture containing 800 ppm of NO, 800 ppm of NH_3_, 3.5 vol.% of O_2_, and He as an inert. The total gas flow was 100 mL·min^−1^, which led to the GHSV of 30,000 mL∙h^−1^∙g^−1^. The NH_3_-SCR tests were carried out in a temperature range of 150 °C to 450 °C with 50 °C as a step. Concentrations of the residual NO and N_2_O (the by-product of the reaction) in the outlet gas were analyzed continuously by the FT-IR detector (ABB 2000, AO series). The results were calculated based on the specific algorithm and collected as an xls file. This manner of the analysis did not provide FT-IR spectra of the post-reaction gas mixture; however, it did enable a relatively fast calculation of the experimental results. The conversion of NO was calculated according to the formula represented by Equation (1):

(1) $$NO_{conv}=\frac{\left(C_{NO_{in}\;}-C_{NO_{out}}\right)}{C_{NO_{in}}}\;\cdot 100\%$$

where $NO_{conv}$—NO conversion, $C_{NO_{in}}$—inlet concentration of NO, $C_{NO_{out}}$—outlet concentration of NO in the gas mixture. 

## 3. Results

### 3.1. Catalytic Tests

#### Catalytic Performance of Fresh HT-Clinoptilolite

**Figure 1:**
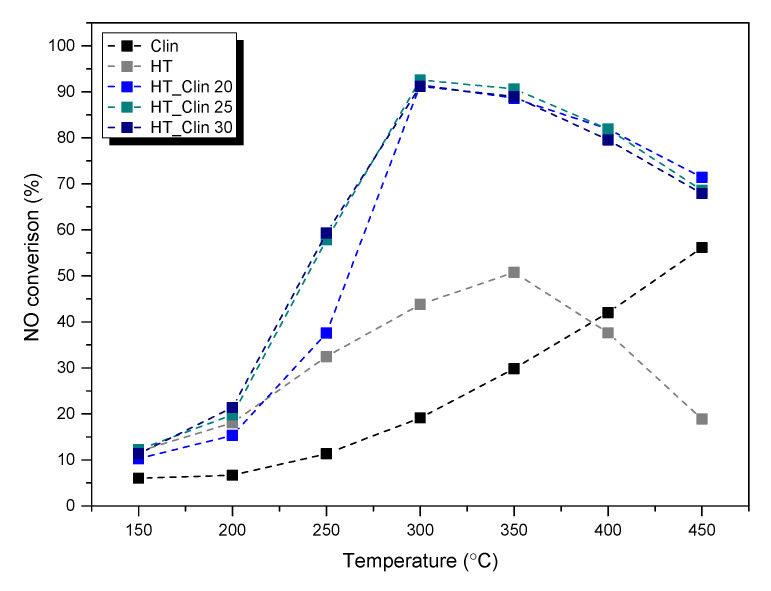
NO conversion obtained for the samples.

**Figure 2:**
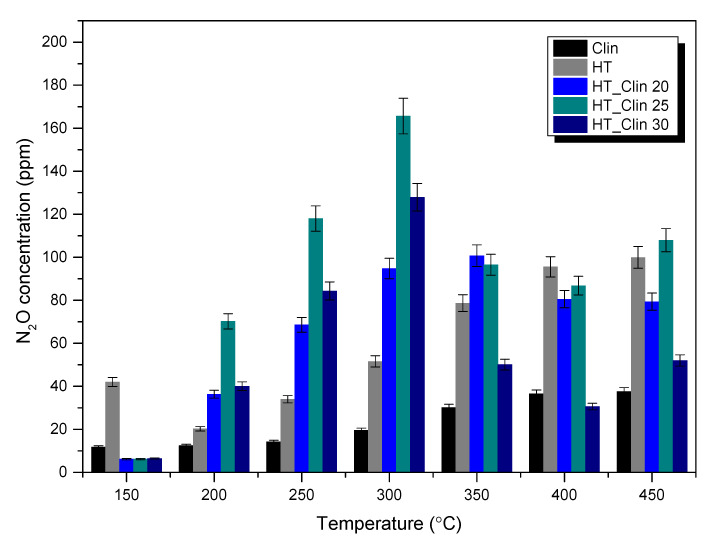
N_2_O concentration emitted during catalytic reaction over the samples.

The samples of hydrotalcites co-precipitated on clinoptilolite were tested as the catalysts of selective catalytic reduction of NO with ammonia. NO conversion obtained for the samples is presented in Figure 1. Furthermore, concentration of N_2_O (as the by-product of NH_3_-SCR) at the reactor outlet is depicted in Figure 2.

It can be observed from Figure 1 that non-calcined, single-phase HT exhibited the lowest NO conversion and that the temperature of 50% NO conversion (*T_50_*) for this catalyst was ca. 350 °C. Additionally, above this value, catalytic activity of the material considerably decreased. On the other hand, around 55% of NO was eliminated at 450 °C for clinoptilolite, even before the introduction of the active phase. Relatively high activity of this material in SCR reactions can be ascribed to the natural presence of iron species in its structure [28]. It can be noticed that combination of clinoptilolite and hydrotalcite phase drastically improved the activity of the single materials in NO removal. The conversion was observed to increase up to 300 °C, reaching the maximum of 95%, independently of the concentration of HT on clinoptilolite. Of interest is that the percentage coverage of the zeolite with LDH phase did not significantly influence the catalytic performance of the materials. NO conversion of HT_Clin 25 and HT_Clin 30 was almost identical in the entire temperature range, while HT_Clin 20 was slightly less active within 200–275 °C. Positive influence of the co-existence of HT- and zeolitic phase on NO elimination resulted most probably from two reasons: (1) clinoptilolite acted during the reaction as the source of Brönsted acid sites, which enabled efficient adsorption of ammonia during the reaction [41], and (2) hydrotalcite phase was a source of Cu^2+^ and Fe^3+^ sites of Lewis type, contributing to low- and high-temperature activity, respectively [42]. Additionally, the catalytic activity of the materials above 300 °C slightly decreased, which can be attributed to non-desired direct oxidation of ammonia. Therefore, the reaction probably dominated over NH_3_-SCR in the high-temperature range and was able to contribute to the production N_2_O [43]. 

The emission of N_2_O (a common by-product of NH_3_-SCR) during the reaction is a very important factor for the potential application of the catalyst in the industry. The selectivity towards the formation of N_2_O is a source of information on the side reactions occurring. It can be noticed from Figure 2 that the concentration of N_2_O for raw clinoptilolite and single-phase hydrotalcite did not exceed 40 and 100 ppm, respectively. In contrast, the yield of N_2_O was drastically elevated for HT_Clin samples. Interestingly, despite very similar activity in NO reduction in the high-temperature range, the emission of N_2_O for the materials was completely different. The increased generation of nitrous oxide was temperature-dependent, since below 300 °C it followed the order of HT_Clin 20 < HT_Clin 30 < HT_Clin 25, while above 300 °C was sorted as HT_Clin 30 < HT_Clin 20 < HT_Clin 25. Besides, one should note that the amount of the emitted N_2_O was almost identical for HT_Clin 20 and HT_Clin 25 within a range of 350–400 °C. What is more, the increase in N_2_O emission was observed only up to 300 °C, while within the range of 300–450 °C concentration of nitrous oxide visibly reduced, especially for HT_Clin 30. The explanation of the emission of N_2_O below 300 °C was proposed by Zhang and Yang [44]. The authors postulated that the source of nitrous oxide in the low-temperature NH_3_-SCR is ammonium nitrate (NH_4_NO_3_), formed by the interaction of NH_3_ and surface nitrates on the catalyst surface at the initial level of the process. Decomposition of NH_4_NO_3_ is followed by reaction (R1):

NH_4_NO_3_ → N_2_O + 2 H_2_O (R1)

High-temperature (300–450 °C) formation of N_2_O may be associated with the Lewis acidic sites originating from Cu and zeolite support. As it was reported elsewhere, the aforementioned acidic sites are prone to form NH from oxidative dehydrogenation of NH3. The former further reacts with NO from feed gases produce N_2_O [45]. Considering the level of NO conversion of the most active catalysts at and above 300 °C, and the amount of the formed N_2_O by-product, such an explanation has its confirmation in the experimental results.

Generally, it can be observed that the coverage of clinoptilolite with HT noticeably influenced concentration of N_2_O above 300 °C. This observation can be explained by the possible decomposition of hydrotalcite phase, taking place between 300 and 350 °C, simultaneously with the catalytic reaction. Thermal treatment of layered doubled hydroxides results in the formation of mixed metal oxides, beneficially distributed within the supporting material [18]. For this reason, it was expected that the presence of zeolite played a crucial role in the advantageous distribution of Fe-Cu oxides, since the reduction of N_2_O emissions with the increasing temperature has not been observed for single-phase HT. Therefore, the generation of mixed oxides on the surface of clinoptilolite could contribute to a higher selectivity of the catalysts to N_2_ and H_2_O above 300 °C.

### 3.2. Characterization of the Catalysts

#### 3.2.1. Chemical Composition and Crystallinity of the Samples

The content of Si, Al, Mg, Fe, and Cu in the samples was measured by inductively coupled plasma-optical emission spectrometry (ICP-OES). The overview of the obtained data, expressed as wt% of each component, is presented in Table 1. It can be observed that the chemical composition of non-supported hydrotalcite is in agreement with the calculated one. Besides, the quantity of Si and Al in the non-modified clinoptilolite, which gives an approximate Si/Al molar ratio of 4.4, corresponds to that reported in the literature [46]. After modification with HCl, concentration of aluminum in the zeolite slightly decreased due to dealumination, which occurs normally after acid treatment [47]. Thereby, the initial modification with HCl resulted in successful, controlled elimination of Al cations from the zeolitic framework. According to Garcia-Basabe and co-workers [47], breaking Al–O bonds and the successful extraction of Al by diluted acids does not affect the topology of the zeolitic framework, however, it does result in a higher thermal stability and pore volume. What is more, Ghasemian et al. [48] reported the improved catalytic performance of clinoptilolite in propane-SCR after the zeolite was leached with mild oxalic acid. The beneficial effect of pre-treatment with acid was ascribed to re-distribution and the increased strength of the acid sites of clinoptilolite. One of the characteristic features of clinoptilolite is the natural presence of iron incorporated into its framework, as confirmed by ICP-OES. Due to that characteristic, the material can exhibit relatively high NO conversion, even in its natural form. 

Deposition of the hydrotalcite phase resulted in a gradual reduction of Si concentration, delivered to the sample exclusively by the zeolitic support. Moreover, the observed decrease was linear with the increasing coverage of clinoptilolite with HT phase. In contrast, the content of Al exhibited reverse trend, as the result of the presence of aluminum in the deposited hydrotalcite and followed the order of HT_Clin_20 < HT_Clin_25 < HT_Clin_30.

**Figure 3:**
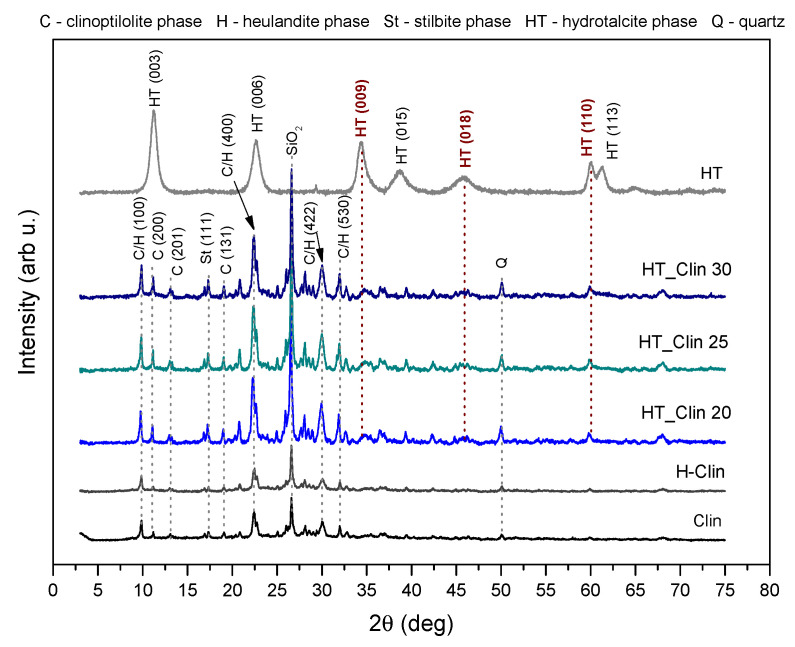
XRD diffractograms obtained for the samples.

XRD patterns obtained for the investigated materials are presented in Figure 3. Non-modified zeolite exhibited diffraction maxima located at the 2*θ* positions of ca. 9.85, 11.18, 17.57, 19.15, 22.31, and 32.91°, which are attributed to characteristics reflections of clinoptilolite (JCPDS#38-0237). Other peaks, confirming that clinoptilolite was the main phase of the natural material ,are located at the 2*θ* values of 22.45, 26.8, 28.4, 30.1° (JCPDS#39-1383) [49,50]. However, the reflections at 9.85, 22.31, 30.02, and 31.81° suggested that some part of the aluminosilicate coexists with the crystalline phase of heulandite [23]. Besides, the diffraction maxima at *2θ* of 16.61, 26.65, and 50.07° indicated the appearance of stilbite and SiO_2_ impurities, respectively [51]. The diffraction pattern of H-Clin showed a similar shape to that of the raw zeolite and the position of the reflections characteristic of heulandite/clinoptilolite phases remained unchanged. 

In the case of the fresh hydrotalcite, a single crystalline phase was detected by the reflections positioned at *2θ* of 11.32, 22.74, 34.43, 38.72, 45.93, 60.03, and 61.44°, corresponding to (*hkl*) planes given in the figure. Therefore, the prepared material is characterized by a multilayered hydrotalcite-like structure (ICDD 00-022-0700) [15]. The absence of other diffraction maxima in the pattern suggested that no secondary phases were formed during the preparation of the material. Since the background signals were not altered and the diffraction peaks attributed to HT were broad, the obtained material was characterized by a small crystallite size [52]. The Fe- and/or Cu-containing phases were not detected in the obtained XRD patterns, suggesting their incorporation into the structure of hydrotalcite. The unit cell parameters were calculated for the HT sample according to the method proposed by Rives [53]. According to the author, the parameter *c* represents three times the distance between the layers and strongly depends on the type and orientation of interlayer anions. It can be calculated using two methods: (1) from the position of the first diffraction maximum, (003), *c* = 3*d*(003) or (2) from the position of three first reflections, (003), (006), and (009), as the sum of *d*(003) + 2*d*(006) + 3*d*(009). In this work, the *c* parameter was calculated according to the manner (2) and was found to be 22.61 Å. Additionally, the *c’* parameter, calculated as *c*/3, enables the researcher to establish the type of anion in the interlayer space. Thereby, the *c’* value of ca. 7.52 Å confirmed the presence of CO_3_^2−^ (where the mean distance between the anions equals 3.3 Å) [1,54]. Furthermore, parameter *a* of the unit cell describes the average cation–cation distances in the hydrotalcite layers, and hence it is strictly correlated with the ionic radius of the cations [15]. The parameter *a* is typically calculated from the first reflection at 2*θ* = 60° (originating from the diffraction by (110) planes) according to the formula *a* = 2*d*(110). The calculated values of *a* = 3.08 Å and *c* = 22.61 Å are in good agreement with those reported in the literature [14,15,54] and suggested that the prepared hydrotalcite exhibited rhombohedral (3R) symmetry. Besides, the crystallite size calculated for HT from Debye–Scherrer formula varied in the range of 100–200 Å. 

In the case of HT-modified clinoptilolite samples, all of the obtained XRD patterns displayed identical shape, regardless of the percentage coverage of the zeolite with HT. Both the position and the intensity of diffraction peaks characteristic of clinoptilolite were identical with that obtained for the non-modified material. Thus, the heulandite framework was not sensitive for the deposition of hydrotalcite phase. However, the appearance of new, relatively broad peaks of low intensity at 2*θ* of ca. 34.43 (009), 45.93 (018), and 60.03 (110)° can be interpreted as the successful formation of a hydrotalcite structure on the surface of clinoptilolite. Additionally, it should be emphasized that at 2*θ* < 25° the diffraction peaks characteristic of clinoptilolite and hydrotalcite could partially overlap with each other due to their presence at similar diffraction angles.

#### 3.2.2. Textural and Surface Properties of the Samples

**Figure 4:**
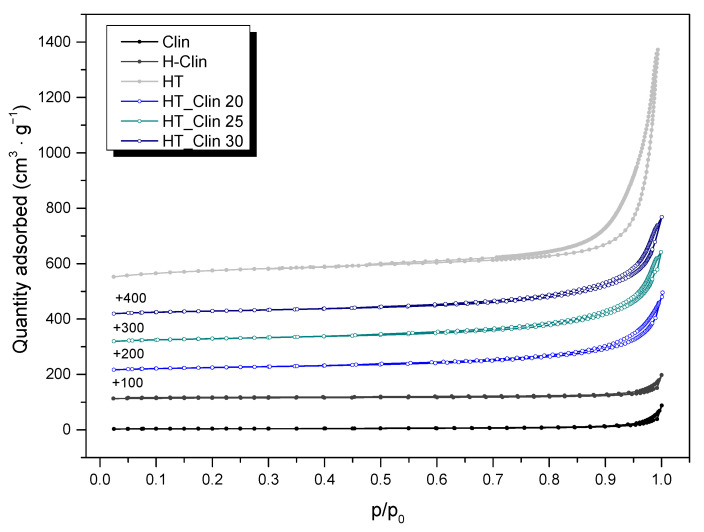
Low-temperature N_2_ sorption isotherms obtained for the samples (for better visibility, the isotherms were shifted by the values given in the figure).

Low-temperature N_2_ sorption was conducted in order to analyze textural features of the investigated materials. The isotherms obtained for non-supported hydrotalcite and HT co-precipitated on natural zeolite are presented in Figure 4. The isotherm of raw clinoptilolite was of type IV (a), with the narrow H3 hysteresis loop, according to the IUPAC classification [55]. Such specification of the hysteresis loop is characteristic of the capillary density in the wedge-shaped mesopores. The monolayer-multilayer adsorption at *p/p*_0_ < 0.6 and capillary condensation in mesopores at *p/p_0_* > 0.85 suggested that the zeolite contains also micropores [23,56]. Additionally, such type of isotherm is characteristic of the materials consisting of non-rigid aggregates of plate-like particles, which is in line with SEM results [55,57]. The isotherm of H-Clin showed similar shape to that of non-modified zeolite, suggesting that the textural parameters of the materials were probably identical. Similarly to clinoptilolite, the isotherm of non-supported hydrotalcite had a shape characteristic of the type IV (a) [55]. Furthermore, the H1 hysteresis loop confirmed the mesoporous texture of the prepared HT, with well-defined and cylindrical pores [15,52]. Low-temperature N_2_ sorption for hydrotalcite co-precipitated on clinoptilolite is expressed by IV type isotherm, classified additionally as a subtype H1, starting at *p/p*_0_ of around 0.8. Therefore, co-precipitation of HT resulted in the formation of additional mesopores of cylindrical-like shape [3]. As summarized in Table 2, the volume of mesopores in the HT_Clin samples increased linearly with the percentage coverage of clinoptilolite with HT. Therefore, the fluent rise of the isotherms obtained for the materials at *p/p*_0_ > 0.85 can be explained by the contribution of new mesopores present in the deposited hydrotalcite-like phase. 

*S_BET_* of the raw clinoptilolite (see Table 2) was 13 m^2^·g^−1^, which is typical for this material [24,58]. Vassileva and Voikova [57] postulated that such a low value of specific surface area can be linked with the adsorption of N_2_ molecules on the external surface of the material, since they cannot enter the internal pores of clinoptilolite. Additionally Sprynskyy and co-workers [59] explained this effect by the presence of exchangable cation complexes, which exist in the micropores of clinoptilolite and hinder diffusion of nitrogen into the inner structure of the zeolite. Alternatively, the authors suggested that the low N_2_ adsorption capacity can be related to the strong interactions between highly polar structural water molecules and micropores of clinoptilolite. Therefore, the volume of micropores determined for the sample is as well very low. After pretreatment with HCl, specific surface area of clinoptilolite increased to 47 m^2^·g^−1^, as the result of decationation and dealumination, both caused by acid leaching. Additionally, removal of pore-blocking species enhanced textural features of the zeolite by the elevation of micro- and mesopore volume, which is in line with higher *S_BET_* of the sample. Non-supported HT showed *S_BET_* of 256 m^2^·g^−1^, which was the highest among the investigated materials, due to well-developed mesoporosity. Moreover, the value was even higher than that reported in the literature for co-precipitated hydrotalcites [15,54,60]. Furthermore, it can be observed that high specific surface area of hydrotalcite contributed to the slight increase in *S_BET_* determined for HT_Clin samples, which varied from 88 to 103 m^2^·g^−1^, depending on the zeolite coverage. This effect can be attributed to the presence of additional mesopores originating from LDH phase.

#### 3.2.3. Morphology of the Samples

**Figure 5:**
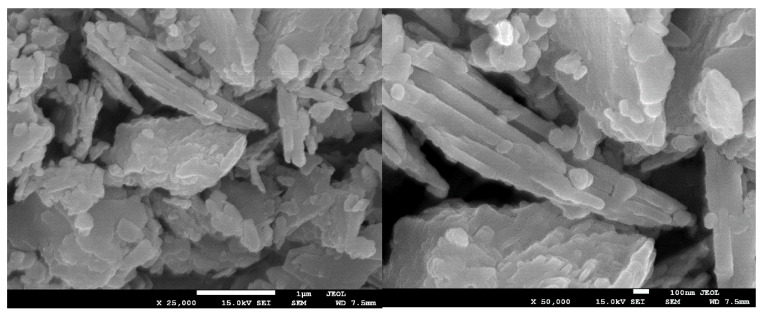
SEM images of non-modified clinoptilolite.

The morphology of clinoptilolite, before and after modification with hydrotalcite and the non-supported LDH, was analyzed by means of scanning electron microscopy (SEM). As shown in Figure 5, the natural clinoptilolite used in the studies exhibited high level of crystallinity. One can notice that the zeolitic structure did not consist of individual grains, but randomly arranged, heterogeneous plate-like layers, peripherally covered with impurities. According to the literature [61], the observed splitting of the separated layers is typical for clinoptilolite. Additionally, such structural characteristics of zeolites is advantageous to obtaining uniform dispersion of the catalytically active species [61,62].

**Figure 6:**
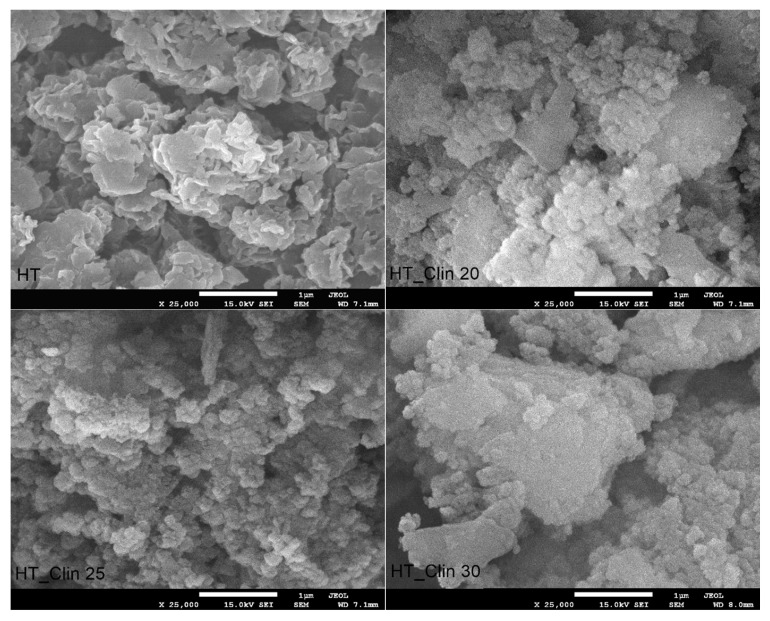
SEM images of non-supported hydrotalcite (HT) and clinoptilolite samples covered with hydrotalcite at different levels of coverage.

Figure 6 shows SEM images of non-supported and clinoptilolite-supported hydrotalcite. It can be observed from the micrograph recorded for HT that the material is characterized by strong adhesion of the individual layers, which are poorly dispersed. Zulkifli and co-workers [63] reported similar structure of hydrotalcite prepared by co-precipitation, comparing it to the “sand-rose”. Zhang et al. [64] explained that this kind of morphology is a consequence of the presence of adsorbed or structural water and non-bridging hydroxyl groups in the interlayer space. Therefore, the plate-like particles of HT can be easily attached to each other through hydrogen bonding. While hydrotalcite phase was co-precipitated on the surface of clinoptilolite, its specification noticeably changed, compared to the non-supported HT. The so-called “sand-rose” morphology of the material was completely lost, and the particles of LDH exhibited uniform shape and size of crystals. What is more, their dispersion on the zeolitic support was strongly correlated with the coverage level. Additionally, one can notice that regardless the amount of co-precipitated HT, its particles were rather clustered on the plate-like particles of clinoptilolite. Interestingly, the highest agglomeration degree was observed for HT_Clin 25, in which the zeolite was almost completely covered with HT crystals. 

#### 3.2.4. Characteristic Chemical Groups in the Samples

**Figure 7:**
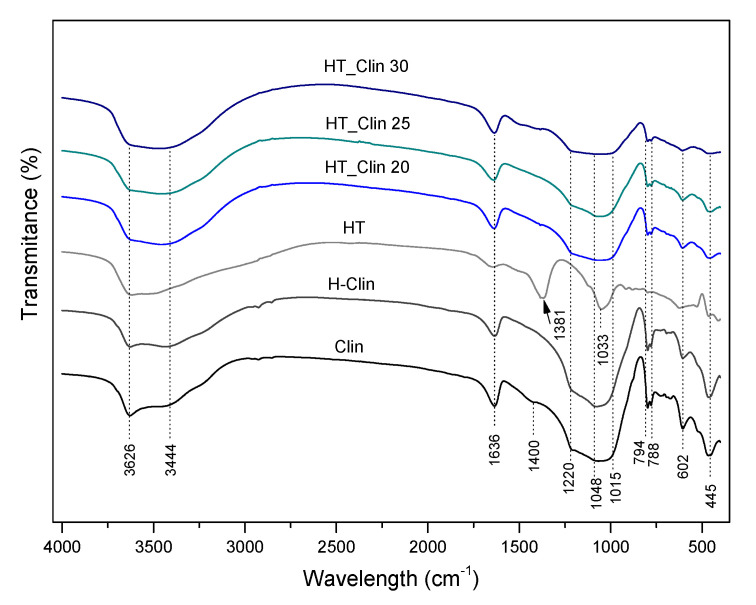
FT-IR spectra recorded for the investigated samples.

The characteristic chemical groups present in the investigated materials were analyzed using FT-IR spectroscopy. Figure 7 shows the spectra recorded in the wavelength range of 4000–400 cm^−1^, which can be divided into three characteristic regions: (1) 3800–3400 cm^−1^, attributed to the O-H stretching vibrations; (2) 1700–700 cm^−1^, corresponding to stretching Si-O and bending Al-Me-OH vibrations (where Me = cation of metal, for example Mg^2+^ or Ca^2+^), and to structural H_2_O molecules; (3) 700–450 cm^−1^, ascribed to pseudo-lattice vibrations of the zeolitic framework or Al-O bonds in hydrotalcites [24,65]. Therefore, the peaks located at 3626 and 3444 cm^−1^, present in all of the obtained spectra correspond to OH groups of water bonded to Me-OH units of hydrotalcite or/and ≡Al-OH-Si≡ in the zeolite [65,66]. According to the literature [66,67], ≡Al-OH-Si≡ surface groups and the framework ≡Al-OH and ≡Si-OH species are ion-exchange sites of the zeolites. Thus, the similar shape of the absorption bands for all of the materials confirmed that the hydrotalcite phase was deposited on the surface of clinoptilolite, and that no ion-exchange phenomena occurred through the performed modification. Additionally, the strong deformation mode at 1636 cm^−1^ corresponds to the presence of adjacent water molecules in the interlayer space of hydrotalcite or H_2_O in the internal structure of clinoptilolite [68,69]. Furthermore, the peak at 1381 cm^−1^, detected only for non-supported hydrotalcite appeared due to the presence of carbonate ions bonded to the hydroxyl surface of HT [65]. The band at 1033 cm^−1^, observable in the spectrum of HT results from the shift of the interlayer CO_3_^2−^ ions to the so-called “free position”, and it y the strong hydrogen bonding between the interlayer H_2_O and hydroxyl sheets of LDH [70]. Interestingly, the peaks corresponding to carbonate ions were absent for all of the HT_Clin samples, and the spectra obtained for clinoptilolite modified with HT phase were similar to those of raw zeolite. The band at 1220 cm^−1^ is attributed to the asymmetric stretching vibrations of the tetrahedral groups (MeO_4_), while that at 1048 cm^−1^ is linked with the asymmetric stretching vibrations, sensitive to the content of Si and Al in the framework [24]. Hence, a slightly lowered intensity of the latter peak in the case of HT_Clin samples probably results from the decrease in Si and Al content in the materials, as confirmed by ICP-OES. The peak at 1015 cm^−1^ evidenced the presence of iron incorporated into the structure of the materials [71,72]. Last, but not least, the absorption bands at 794, 788, and 602 cm^−1^ appeared due to the asymmetric stretching vibrations of O-Me-O (where Me = Si or Al) [24]. Additionally, the peak at 445 cm^−1^, detected for all of the obtained spectra, can be attributed to the bonds of Al-OH [24,65]. 

#### 3.2.5. Thermal Stability of the Samples

**Figure 8:**
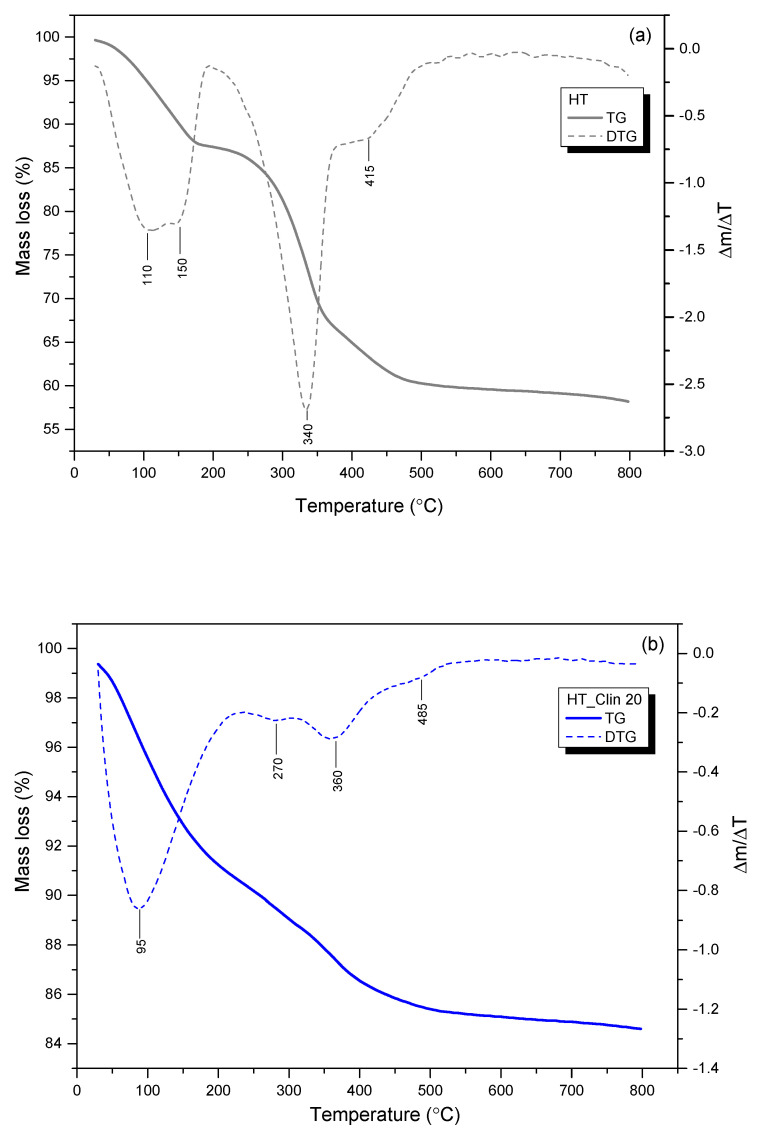

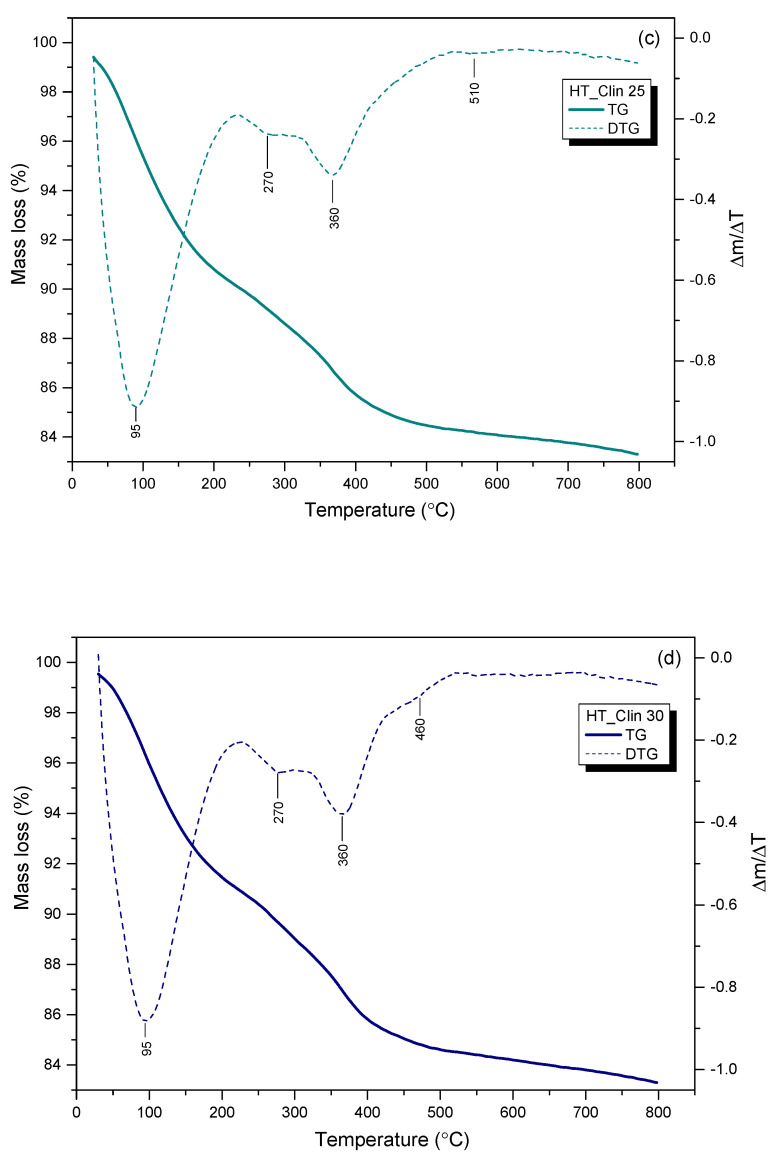
TGA profiles recorded for non-supported hydrotalcite (**a**) and hydrotalcite co-precipitated on the surface of clinoptilolite within 30–800 °C: HT_Clin 20 (**b**), HT_Clin 25 (**c**), HT_Clin 30 (**d**).

Thermogravimetric analysis was carried out to determine the number of steps and the temperature range of the weight loss during thermal treatment of the materials. The thermograms recorded for clinoptilolite-supported and single-phase HT are shown in Figure 8, while the percentage weight losses in the corresponding temperature regions are collected in Table 3. In general, the weight of the materials decreased during thermal treatment by the value of 15–43 wt%. One should note that the initial weight decreased by ca. 0.5 wt.% during equilibration at 30 °C in air. As presented in Figure 8a, thermal treatment of the single-phase hydrotalcite in the temperature range of 30–800 °C resulted in its gradual weight loss of ca. 41.5 wt%, which corresponds to that reported by Chmielarz et al. [14] for Co- and/or Cu-hydrotalcites. The behavior of the sample at the elevating temperature was characterized by two steps: (1) non-isothermal dehydration of hydrotalcite and elimination of small amount of weakly bonded CO_2_ within 30–200 °C and (2) removal of hydroxyl groups from the brucite-like layers and decomposition of volatile anions, such as carbonate between 200 and 400 °C [38,54]. Therefore, the peaks at 110 and 150 °C are related to the release of loosely, structurally and hydrogen-bonded H_2_O from the interlayer space. Furthermore, the stepwise decarbonation of the material is confirmed by the peak at 340 °C (isothermal) and in the region above 415 °C (non-isothermal) [73]. As depicted in Figure 8b–d and detailed in Table 3, the weight loss of HT_Clin samples was significantly lower than that of the pure HT. Thus, the zeolitic support could support the stability of hydrotalcite. However, this phenomenon could also result from lower amounts of HT being subjected to the decomposition compared to the single-phase LDH. It can be observed from the TG/DTG profiles of HT_Clin samples that the temperature of non-isothermal interlayer H_2_O elimination was shifted to 95 °C. This effect could be caused by the simultaneous removal of weakly bonded water molecules from clinoptilolite and the deposited HT phase. Furthermore, regardless of the coverage with hydrotalcite, all of the samples showed similar thermal behavior. Slightly lower values of the total weight loss recorded for HT_Clin 20 might be due to its lower concentration on the surface of clinoptilolite. The peaks appearing in the DTG curves at 270 and 360 °C correspond to the quasi-isothermal dehydroxylation from Al(OH)_3_ and Mg(OH)_2_, respectively. Besides, decomposition of the interlayer carbonates took place between 395 and 410 °C [38,73]. Since the temperature range attributed to the decomposition of CO_3_^2−^ was similar for all of the samples, it can be assumed that the stabilization of the carbonates in the co-precipitated HT was almost identical, regardless of the coverage degree. Last but not least, the peaks of very low intensity, located at 485, 510, and 460 °C for HT_Clin 20, 25, and 30, respectively, could be correlated with the presence of very small amounts of residual nitrates used for HT co-precipitation [38]. All in all, in our case, thermal decomposition of hydrotalcites resulted in the formation of the phase of mixed oxides of Fe and Cu, and the successive collapse of the LDH structure [15,73]. However, it should be noted that the temperature regions of the above-mentioned phase transitions are correlated with the chemical composition of HT (type of cations and interlayer anions) and may be varied for various types of LDHs. To sum up, the weight loss of the samples was related to dehydration, dehydroxylation of the brucite-like structure of hydrotalcite, elimination of weakly bonded CO_2_, and thermal decomposition of volatile anions, such as CO_3_^2−^. 

#### 3.2.6. Speciation of Metals in the Samples 

**Figure 9:**
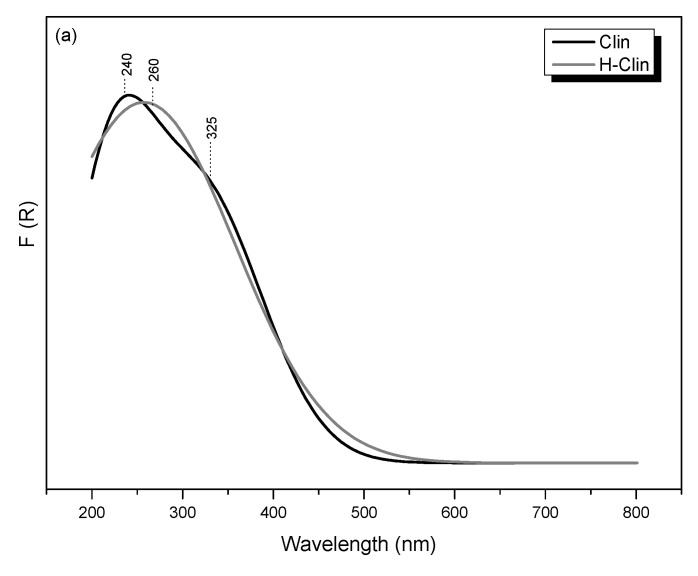

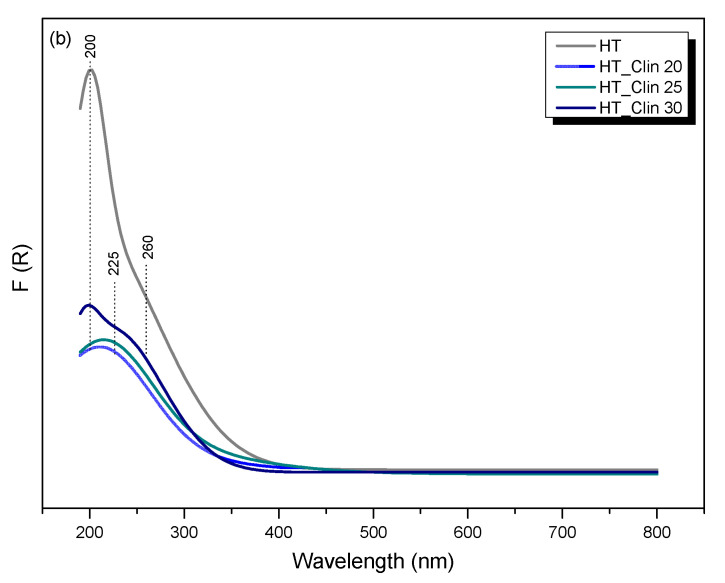
UV-Vis-DRS spectra recorded for (**a**) raw and protonated clinoptilolite and (**b**) non-supported and clinoptilolite-supported hydrotalcite.

UV-Vis-DR spectroscopic studies enabled us to analyze speciation and distribution of transition metals present in natural zeolite or introduced onto clinoptilolite’s surface via hydrotalcite co-precipitation. Figure 9a shows UV-Vis-DR spectra recorded for the parent and hydrogen forms of clinoptilolite. It can be observed that the raw clinoptilolite exhibited two strong absorption bands, which confirmed the presence of iron incorporated into the zeolitic structure [47]. The first band, centered at around 240 nm is attributed to ligand-to-metal charge transfer (LMCT, O^2−^ → Fe^3+^) transitions of isolated Fe^3+^ species, tetrahedrally coordinated in the extra-framework positions [74]. It can be observed that distribution of iron in the zeolite slightly changed after protonation, since the first absorption maximum was shifted to 260 nm. This result is likely caused by oligomerization of Fe^3+^ in the octahedral positions and formation of extra-framework Fe_x_O_y_ species, primarily existing in the form of dinuclear complexes, such as [HO-Fe-O-Fe-OH]^2+^ [75]. A similar effect was observed by Chemielarz et al. [43] for acid-modified vermiculite. Furthermore, the presence of oligonuclear Fe_x_O_y_ was confirmed by the band at 325 nm, present in the spectra of both Clin and H-Clin samples. The very weak and broad signal, detected in the spectra above 400 nm, is associated with aggregated Fe_2_O_3_ particles located near the pore openings or on the zeolite surface [74,76]. 

The UV-vis-DR spectra obtained for clinoptilolite-supported and single-phase hydrotalcite are presented in Figure 9b. The very intense absorption maximum at 200 nm, observed in the spectrum of HT, corresponds to the charge transfer from oxygen O^2−^ to the monomeric Cu^2+^ ions and tetrahedral Fe^3+^ cations. Additionally, the existence of [Cu^2+^-O^2−^-Cu^2+^] and Fe_x_O_y_ oligomers contributed to the increased intensity of the band within 250–350 nm [76]. The flat shape of the spectrum of HT suggested that transition metals were present in the sample exclusively in the form of isolated cations and oligomers and no other phases were formed, which is in agreement with XRD studies. Co-precipitation of HT on the surface of clinoptilolite resulted in a significant decrease in the absorption bands’ intensity, which was linear to the coverage of the zeolite with hydrotalcite phase. Such a phenomenon could be caused by the fact that clinoptilolite was the major phase of the analyzed materials. It was observed that the position of the absorption maxima corresponded to that of HT. However, the presence of a narrow peak in the spectrum of HT_Clin 30 located at 200 nm and absent for the other HT_Clin samples suggested that the material with the highest concentration of hydrotalcite phase is the most abundant in the isolated species of Fe and Cu. Moreover, the broad bands appearing for all of the materials in the region of 250–325 nm confirmed that hydrotalcite phase probably did not interact with the zeolitic framework and formed a uniform, oligomeric layer consisting of oxygen and transition metals.

## 4. Discussion

The results presented in this work indicate that new NH_3_-SCR catalysts can be successfully prepared by co-precipitation of hydrotalcite phase on natural zeolite. However, there are some important aspects, which considerably influenced satisfactory catalytic performance of such materials.

Chemical composition of the materials is one of the most important aspects which influences their catalytic performance. In the investigated HT_Clin samples, the mass ratio of Fe:Cu was maintained on the approximate level of 2:1 and the loading of the catalytically active metals increased following the order of HT_Clin 20 < HT_Clin 25 < HT_Clin 30, which was in line with the increasing activity in NH_3_-SCR. According to the literature [28,33,77] the excessive loading of Fe in the catalysts leads to the agglomeration of the active phase, the formation of catalytically inactive Fe_2_O_3_, pore clogging, and decreased activity in the low-temperature range. In contrast, higher loading of Cu improves NO conversion below 300 °C due to the presence of CuO species [78]. Nevertheless, Wang et al. [79] found that, while Cu content is higher than 1.5 wt%, high-temperature activity drastically decreases. Thereby, it is expected that relatively high conversion of NO obtained for HT_Clin samples above 300 °C resulted mainly from well-optimized concentrations of Fe and Cu in the catalysts.

Furthermore, one of the crucial factors contributing to high NO conversion is the choice of appropriate support. The supporting material aims to provide high surface to separate catalytically active species and prevent the formation of big crystals. Considering NH_3_-SCR, specifically porous supports are required, in order to supply the catalyst with sufficient space for the reaction to proceed. In our study, we used a natural zeolite, clinoptilolite. The material is not only relatively cheap, but also exhibits very promising catalytic features, such as the presence of iron incorporated into its framework [51]. Our studies confirmed that application of clinoptilolite as the supporting material for LDH phase significantly increased NO conversion above 250 °C. The strong relationship between the presence of the zeolite and the improved elimination of nitrogen oxide can be correlated with Si-O(H)-Al groups, present in the support. The hydroxyl bridges, tetrahedrally coordinated Si-OH and Al-OH, are considered as the source of Brönsted acid sites, which act as the reservoir of NH_3_ during NH_3_-SCR [80]. Thereby, the presence of zeolitic framework facilitated adsorption of ammonia and consequently, the proceeding of high-temperature Eley–Rideal mechanism, which assumes adsorption NH_3_ and its reaction with NO_x_ from the gas phase [81]. Besides, the acidic character of zeolites is strongly correlated with the Si/Al molar ratio [82]. According to Grajciar and co-workers [80], high-silica zeolites exhibit the lowest stability of Si-O(H)-Al sites, resulting in poor Brönsted acidity. Since the Si/Al molar ratio of clinoptilolite used in this study was relatively low, the enhanced activity of the supported samples could have resulted not only from the presence, but also from the characteristics of the Brönsted centers. Therefore, we concluded that in the case of HT_Clin catalysts, introduction of acid sites was one of the crucial factors determining a high conversion rate of NO. The presence of Cu and Al-O centers delivered to the system from thermal decomposition of hydrotalcite, similarly to the applied zeolite support, were however, a source of Lewis acidic sites, which are considered to favor beforementioned Eley–Rideal mechanism of nitrogen formation [83].

The presented studies indicated that the concentration of the active phase deposited on clinoptilolite did not influence significantly on the catalytic activity. All of the investigated materials exhibited NO conversion characteristic of bimetallic Fe-Cu catalysts, in which CuO contributes to low-temperature activity, while Fe improves high-temperature NO reduction [84,85]. As confirmed by ICP-OES, the concentration of iron in HT_Clin catalysts was higher than that of copper. Therefore, it can be assumed that the loading of Cu in the samples was too low to form sufficient amount of CuO aggregates and increase low-temperature NO reduction. In contrast, the superior high-temperature activity of HT_Clin catalysts can be correlated with the abundance of oligomeric iron and monomeric copper species in the catalysts. According to the literature [86,87,88,89], the metallic active sites in Fe-zeolites show strong inhibition effects by NH_3_ adsorption at low temperatures. Generally, there are two possible explanations for this phenomenon. First of all, ammonia molecules are strongly bonded to Fe centers at low temperatures, enabling coordination of nitrogen oxides on the active sites. Alternatively, and more likely, adsorption of NH_3_ results in the reduction of Fe^3+^ to Fe^2+^, which inhibits the Fe^3+^ → Fe^2+^ redox reaction and oxidation of NO during the catalytic cycle [84]. On the other hand, Brandenberger et al. [90] attributed high-temperature activity of Fe-zeolite catalyst to the presence of dimeric and oligomeric iron species. UV-Vis studies performed over the analyzed HT_Clin catalysts indicated the presence of such structures, regardless of the loading of co-precipitated LDHs. Nonetheless, thermal decomposition of the hydrotalcite phase during the catalytic reaction most likely resulted in the partial redistribution of the active phase. For this reason, we performed additional UV-Vis experiments over HT and HT_Clin catalysts calcined in air. The obtained results, presented in Figure S2, enabled us to predict the type of species produced by the materials during catalytic reaction. It can be observed that thermal treatment indeed changed the distribution of the active phase. Firstly, all of the absorption bands were shifted to a higher wavelength range, compared to the non-calcined samples. The spectrum of HT after calcination exhibited two strong absorption peaks located at 220 and 270 nm, which resulted from the presence of iron oxide [91] and copper oxide [92] nanoparticles, respectively. Furthermore, the intensity of the absorption bands detected for the calcined HT_Clin catalysts was significantly decreased. The spectra of both HT_Clin 20 and 25 were almost identical and differed slightly from that of HT_Clin 30, which confirms that speciation of the active phase was influenced by HT loading. Specifically, the absorption peak appearing for HT_Clin 30 suggested that the sample was the most abundant in oligomeric iron species. What is more, the flat shape of the spectra recorded for HT_Clin catalysts above 500 nm confirmed the absence of bulky particles of metal oxides. Hence, calcination of the hydrotalcite phase produced exclusively extraframework metallic sites (mono- and oligomeric) on the zeolitic support, which had a positive impact on their NH_3_-SCR activity within 200–450 °C. Importantly, it was found that the oligomeric iron sites not only contribute to high-temperature activity, but are also active in non-selective oxidation of NH_3_ above 350 °C [90]. This postulate can partially explain the highest concentration of N_2_O observed for HT, HT_Clin 20 and 25. However, one should note that the emission of nitrous oxide was not very pronounced for HT_Clin 30. Furthermore, Pereda-Ayo and co-workers [85] analyzed the behavior of different copper species during NH_3_-SCR reactions. The authors found that Cu^2+^ ions, likely formed in low-copper content catalysts, maintain high NO conversion above 300 °C. Considering the relatively low loading of Cu in HT_Clin catalysts and the obtained results of UV-Vis experiments, the most satisfactory catalytic performance of the examined materials within 300–350 °C resulted not only from the presence of polynuclear Fe_x_O_y_, but also monomeric Cu^2+^ species. 

Thermal decomposition of hydrotalcite phase during NH_3_-SCR can be confirmed by monitoring of CO_2_ emissions in the post-reaction gas mixture. As presented in Figure S1, the concentration of carbon dioxide produced during the reaction over non-modified clinoptilolite was very low, or even below the detection limit of the instrument. Therefore, CO_2_ appeared in the exhausts exclusively due to the decomposition of the hydrotalcite phase. In the case of HT and HT_Clin 20, the emission of CO_2_ was maintained on a similar level, regardless temperature of the reaction. In contrast, for HT_Clin 25 and HT_Clin 30 the production of carbon dioxide reached its maximum at 300 °C and moderately decreased above this temperature. The observed phenomenon was linked with the decomposition of the interlayer carbonates, which takes place at approximately 300 °C, as confirmed by TG experiments. It is of considerable interest that the temperature of the highest emission level of CO_2_ corresponds to that of the greatest yield of N_2_O. Therefore, the presence of carbon dioxide in the reacting gas mixture limited selectivity to the desired reaction products, especially in the case of HT_Clin 25 and 30. One possible explanation of this effect may be found in the kinetic diameters of the molecules present in the gas mixture. Since an ammonia molecule is smaller than a CO_2_ molecules, carbon dioxide could set some diffusion limits for NH_3_ to be successfully adsorbed on the catalyst surface. However, more detailed research is required to determine the impact of the presence of CO_2_ on the selectivity of HT_Clin catalysts to nitrous oxide. 

In contrast to NO conversion, co-precipitation of hydrotalcite had a noticeable impact on the concentration of the emitted N_2_O. In general, there are a few varied reasons for such phenomenon. First of all, it was observed that the generation of nitrous oxide was higher for HT_Clin samples compared to HT in the entire temperature range. As proposed by Zhang and Yang [44], generation of nitrous oxide below 300 °C results from thermal decomposition of NH_4_NO_3_. Furthermore, Gao et al. [84] found that Brönsted and Lewis acidity promotes formation of ammonium nitrate on the zeolite-supported catalysts in the low-temperature region of NH_3_-SCR, which is followed by the reactions (R2–R4):

H^+^ + NH_3_ → NH_4_^+^ (R2)

NH_4_^+^ + HNO_3_ → NH_4_NO_3_ + H^+^ (R3)

L ··· NH_3_ + HNO_3_ → NH_4_NO_3_ + L (L—Lewis acid site) (R4)

Therefore, the introduction of acid sites originating from the zeolitic support not only promotes the conversion of NO, but also contributes to non-desired production of N_2_O. The increased concentration of N_2_O in the residual gas mixture can be also correlated with the competitive ammonia oxidation taking place on copper species [14]. Nevertheless, when the temperature of the reaction exceeded 300 °C, the concentration of nitrous oxide gradually decreased. Feng and co-workers [41] found that decomposition of NH_4_NO_3_ is promoted by Brönsted acid sites, which participate in several steps of the reaction. Therefore, depending on the temperature region, introduction of acid sites originating from clinoptilolite could indirectly influence the N_2_O yield in different manners. Alternatively, the decreasing concentration of nitrous oxide above 300 °C can be linked with decomposition of hydrotalcite phase and formation of dispersed metal oxides, which limited the production N_2_O [93]. What is more, iron zeolites were confirmed to be active catalysts of N_2_O decomposition [74,94,95,96]. Pérez-Ramírez et al. [96] found that the extra-framework oligonuclear iron species exhibit superior activity in the reaction compared to the isolated Fe^3+^. The authors ascribed this effect to higher affinity to oxygen, which is crucial fordirect decomposition of N_2_O. Likewise, Sun et al. [74] suggested that more clustered iron sites are required to provide surface oxygen for the reaction with gaseous N_2_O. Considering the cited studies, decomposition of the hydrotalcite phase during the catalytic reaction and generation of oligomeric iron species (see Figure S2) promoted partial decomposition of N_2_O. Nevertheless, despite all of the samples containing dimeric and oligomeric iron sites, one can notice that the decreased concentration of nitrous oxide in the high-temperature range was mostly pronounced for HT_Clin 30. Therefore, the active centers of HT_Clin 20 and 25 promoted oxidation of NH_3_ to nitrous oxide rather than decomposition of the generated by-product. Another possible explanation of the lower N_2_O yield observed for HT_Clin 30 is the superior textural parameter possessed by the catalyst. As confirmed by N_2_ sorption studies, the sample exhibited the highest mesopore volume among the materials, which improved the diffusion of the reacting molecules. What is more, a relatively high value of *S_BET_* of the sample facilitated dispersion of the metallic active centers. In fact, it can be observed that the single-phase HT sample showed even better textural characteristics than HT_Clin 30. Hence, NH_3_-SCR activity of HT was probably limited by the absence of supporting material, abundant in acidic centers, which can adsorb and accumulate ammonia molecules. Given these points, the deposited hydrotalcite phase and clinoptilolite structure acted synergistically, forming new, promising, and ecologically friendly catalysts for the reduction of NO via NH_3_-SCR.

The catalytic features of the investigated materials can be also evaluated basing on the comparison of their performance in NH_3_-SCR with other catalysts. Therefore, in Table 4 we have summarized NO reduction and N_2_O concentration obtained for various catalysts tested in the conditions similar to that applied in our study. In fact, low-temperature activity of the catalysts consisting of HT and clinoptilolite is lower compared to Fe-zeolites of MWW family and single-phase Co-Cu mixed oxides derived from LDH. However, in the case of HT_Clin 25 and 30, NO conversion at 200 °C was very similar to that of Fe-clinoptilolite prepared by post-synthesis method. The lower activity of the investigated materials is most likely caused by three reasons: (1) speciation of the active phase, (2) chemical composition of the samples, (3) omitting of the calcination step. Higher levels of low-temperature activity of the catalysts supported on MWW zeolites resulted from the presence of isolated Fe^3+^ species in the framework and extraframework positions. Additionally, Mg^2+^ cations, originating from hydrotalcite phase in HT_Clin samples, could partially hinder the adsorption of NH_3_ at low temperatures and inhibit the catalytic reaction. Furthermore, better catalytic performance of mixed metal oxides obtained from LDH could be a consequence of higher loading of Cu and the fact that the material was calcined prior to the catalytic reaction. However, the abundance of copper in the cited catalyst led to significant decrease in high-temperature NO conversion, caused by side reactions of NH_3_-SCR catalyzed by Cu above 350 °C. Thereby, it is expected that the controlled introduction of copper into HT_Clin catalysts was beneficial to maintain satisfactory high-temperature activity. Last, but not least, clinoptilolite modified with Fe by standard, post-synthesis method showed similar catalytic performance to HT_Clin 25 and HT_Clin 30. Since Fe loading in the cited Fe-Clin catalyst was approximately 5 wt%, lower activity of HT_Clin 20 results from the content of the active phase in the sample. What is more, one should note that emission of N_2_O for the HT_Clin catalysts exceeded that of other materials summarized in Table 4. Hence, further research is needed to improve selectivity of the catalysts and hinder side-reactions of NH_3_-SCR by the optimization of the chemical composition and preparation procedure.

## 5. Conclusions

The presented study offers one of the first fundamental investigations into the synergistic effect between hydrotalcite-derived active phase and zeolitic support of NH_3_-SCR catalyst. We have presented a novel approach for the synthesis of NH_3_-SCR catalysts, and it was found that natural clinoptilolite improves distribution of the metallic active centers originating from hydrotalcite. Moreover, it supplies the prepared dual-phase catalytic system with the acid sites, which actively adsorbs ammonia during the reaction. As a consequence, the supported LDHs showed superior catalytic activity in NO reduction over the single-phase hydrotalcite. Additionally, we reported that the coverage of clinoptilolite with HT did not influence the conversion of NO. It did, however, affect the emission of N_2_O. The generation of nitrous oxide below 300 °C probably resulted from side reactions of NH_3_-SCR. Conversely, within the range 300–350 °C, it was ascribed to the decomposition of ammonium nitrates or competitive oxidation of NH_3_ promoted by copper species. The presented experiments also indicated that calcination of hydrotalcite phase co-precipitated on the natural zeolite prior to NH_3_-SCR test is not required to obtain satisfactory NO conversion and can be conducted during the catalytic reaction. Since our results are encouraging, the most active samples should be tested under various reaction conditions (i.e., without NH_3_, with SO_2_ present in the reacting gas, or using various concentrations of the components of the gas mixture). Thereby, our future studies on the clinoptilolite-hydrotalcite catalysts will concentrated on this point.

## Figures and Tables

**Table 1:** Chemical composition of the materials determined by ICP-OES.

Sample Code	Si (wt%)	Al (wt%)	Mg (wt%)	Fe (wt%)	Cu (wt%)	Si/Al
HT	0.16	6.20	21.42	5.24	4.47	-
Clin	32.11	6.89	0.38	1.10	-	4.4
H-Clin	32.71	5.89	0.24	0.99	-	5.3
HT_Clin 20	27.52	5.62	4.19	1.78	0.81	4.7
HT_Clin 25	26.25	5.70	4.99	1.94	0.97	4.5
HT_Clin 30	25.95	5.86	5.67	2.09	1.02	4.2

**Table 2:** Structural and textural parameters of the investigated samples.

Sample Code	S_BET_^a^ (m^2^·g^−1^)	Micropore Volume ^b^ (m^3^·g^−1^)	Mesopore Volume ^c^ (m^3^·g^−1^)	Mean Pore Diameter ^d^ (nm)
HT	256	0.027	1.35	21
Clin	13	0.003	0.059	18
H-Clin	47	0.016	0.120	7
HT_Clin 20	88	0.002	0.393	14
HT_Clin 25	103	0.002	0.495	18
HT_Clin 30	102	0.002	0.523	17

^a^ Calculated from Brunauer–Emmett–Teller (BET) equation. ^b^ Determined by t-plot method. ^c^ Calculated from the Barrett–Joyner–Halenda (BJH) desorption volume of mesopores. ^d^ Average pore diameter (4V/A by BET equation).

**Table 3:** Approximate weight loss recorded for the materials during TG analysis.

Sample Code	Weight Loss in the Temperature Range (wt%)			
	30–200 °C	200–375 °C	375–800 °C	Total
HT	12.0	22.5	7.0	41.5
HT_Clin 20	8.5	3.5	0.5	12.5
HT_Clin 25	9.0	4.5	3.0	16.5
HT_Clin 30	8.0	5.5	3.0	16.5

**Table 4:** Comparison of the catalytic performance of HT-Clin samples with other catalysts tested under similar reaction conditions.

Sample Code	NO Conversion (%) ^a^	N_2_O Concentration (ppm) ^a^	Composition of the Gas Mixture	GHSV (ml·h^−1^·g^−1^)	Ref.
HT	32|51	34|79	800 ppm NH_3_ 800 ppm NO 3.5 vol% O_2_ He as an inert	30,000	This work
HT_Clin 20	37|88	69|101			This work
HT_Clin 25	57|89	118|97			This work
HT_Clin 30	58|91	84|50			This work
Fe-MCM-22 ^b^	100|87	<10			[33]
Fe-MCM-36 ^b^	100|90	<10			[32]
Fe-ITQ-2 ^b^	95|78	<10			[32]
Co_0.08_Cu_0.16_^c^	80|60	<10|70			[15]
Fe-Clin ^d^	64|100	65|75			[27]

^a^ NO conversion and N_2_O concentration at 250 and 350 °C, respectively. ^b^ Catalysts prepared by one-pot synthesis method with Fe loading of approximately 5 wt%. ^c^ Catalyst prepared by co-precipitation of hydrotalcite, followed by calcination at 500 °C in which Cu = 11.9 wt%, Co = 5.8 wt%, and Mg = 14.6 wt%. ^d^ Catalyst prepared by co-precipitation of hydrotalcite, followed by calcination at 500 °C in which Cu = 11.9 wt%, Co = 5.8 wt%, and Mg = 14.6 wt%.

## Data Availability

Data are contained within the article.

## Supplementary Materials

The following supporting information can be downloaded at: https://www.mdpi.com/article/10.3390/ma15227884/s1, Figure S1: Concentration of CO_2_ emitted during NH_3_-SCR performed over the investigated samples; Figure S2: UV-Vis spectra recorded for the calcined catalysts.
