# Supplementary material for: Hydrotalcite-Modified Clinoptilolite as the Catalyst for Selective Catalytic Reduction of NO with Ammonia (NH3-SCR)
Source: Materials (Basel). 2022 Nov 8;15(22):7884. doi: 10.3390/ma15227884 (PMC9696415; doi:10.3390/ma15227884)
Supplement: Supplementary file 1 [file materials-15-07884-s001.zip › materials-1997033-supplementary.pdf]

Hydrotalcite-modified Clinoptilolite as the Catalyst for Selective Catalytic Reduction of NO with Ammonia (NH_3-SCR)

Agnieszka Szymaszek-Wawryca [1,*], Paulina Summa [1], Dorota Duraczyńska [2], Urbano Díaz [3], Monika Motak[1]

Supporting Information

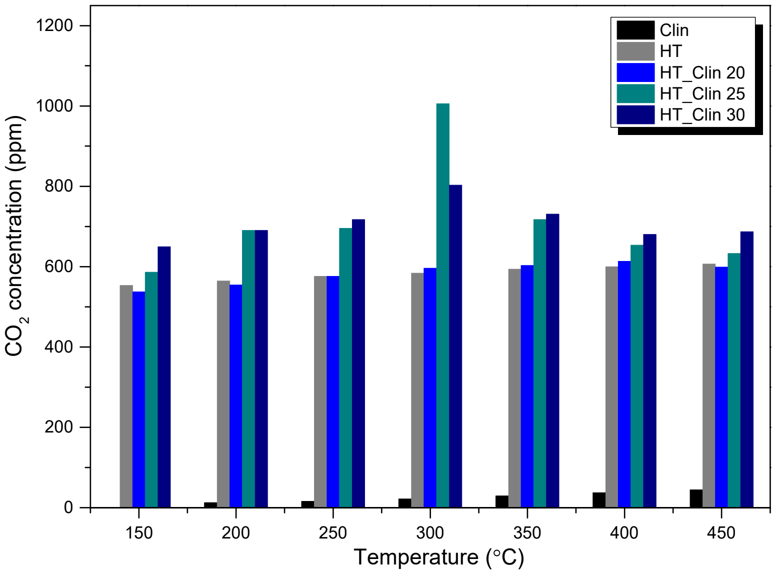

Figure S1. Concentration of CO_2 emitted during NH_3-SCR performed over the investigated samples.

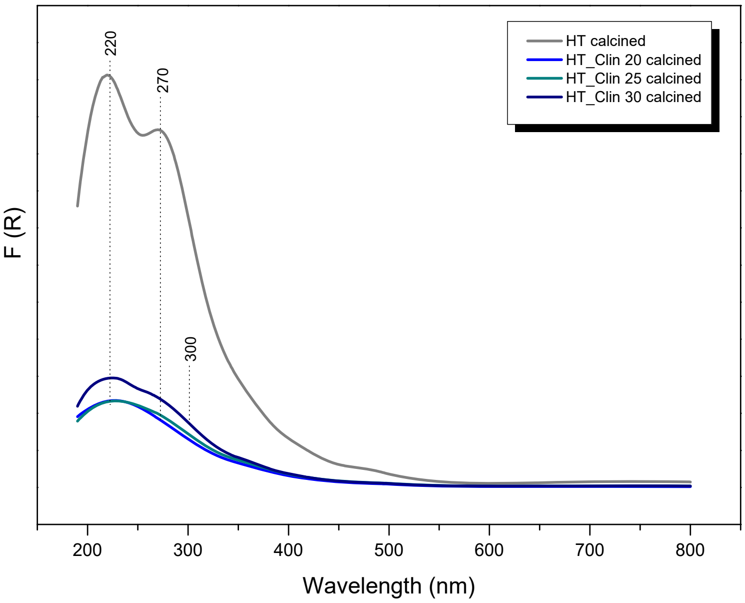

Figure S2. UV-Vis spectra recorded for the calcined catalysts.
